# Admission Cell Free DNA as a Prognostic Factor in Burns: Quantification by Use of a Direct Rapid Fluorometric Technique

**DOI:** 10.1155/2014/306580

**Published:** 2014-06-22

**Authors:** Yaron Shoham, Yuval Krieger, Zvi H. Perry, Gad Shaked, Alexander Bogdanov-Berezovsky, Eldad Silberstein, Amiram Sagi, Amos Douvdevani

**Affiliations:** ^1^Plastic and Reconstructive Surgery Department and Burn Unit, Soroka University Medical Center and Ben-Gurion University of the Negev Faculty of Health Sciences, Beer Sheva 8410100, Israel; ^2^Surgery A Department, Soroka University Medical Center and Ben-Gurion University of the Negev Faculty of Health Sciences, Beer Sheva 8410100, Israel; ^3^Surgery B Department, Soroka University Medical Center and Ben-Gurion University of the Negev Faculty of Health Sciences, Beer Sheva 8410100, Israel; ^4^Clinical Biochemistry Department, Soroka University Medical Center and Ben-Gurion University of the Negev Faculty of Health Sciences, Beer Sheva 8410100, Israel

## Abstract

*Background*. Despite great advances in the treatment of burn patients, useful prognostic markers are sparse. During the past years there has been increasing interest in circulating plasma cell free DNA as a potential marker for tissue injury. We have developed a rapid direct fluorescent assay for cell free DNA quantification that allows obtaining accurate, fast, and inexpensive measurements. *Objective*. To use this technique for measuring plasma cell free DNA levels in burn patients and to further explore the use of cell free DNA as a potential marker of patient outcome in burns. *Methods*. Cell free DNA levels obtained from 14 burn victims within 6 hours of injury and 14 healthy controls were quantified by a direct rapid fluorometric assay. *Results*. Patient admission cell free DNA levels were significantly elevated compared with that of controls (1797 ± 1523 ng/mL versus 374 ± 245 ng/mL, *P* = 0.004). There are statistically significant correlations between cell free DNA admission levels and burn degree (Spearman's correlation = 0.78, *P* = 0.001), total body surface area (Spearman's correlation = 0.61, *P* = 0.02), and total burn volume (Spearman's correlation = 0.64, *P* = 0.014). *Conclusions*. Admission cell free DNA levels can serve as a prognostic factor in burns and future routine use can be made possible by use of our direct rapid fluorometric assay.

## 1. Introduction

Despite great advances in the treatment of burn patients, useful prognostic markers are sparse. During the past years, there has been increasing interest in circulating plasma cell free DNA (CFD) levels, that is, nucleic acids that originate from cell death by apoptosis or necrosis and circulate in peripheral blood, as a potential marker for tissue injury. Release of DNA into the circulation makes it a useful, albeit nonspecific marker of tissue injury. Increased levels of CFD have been detected in many pathological situations such as infection, inflammation, trauma, respiratory insufficiency, pulmonary embolism, autoimmune disease, sepsis, and cancer and have been found to be an adverse prognostic marker for morbidity and mortality [1–6]. 

CFD levels have been studied as a potential marker in burns. Chiu et al. examined CFD levels obtained from 28 burn patients within 24 hours of admission. They found that CFD levels were elevated and related to hospitalization length and to the total body surface area (TBSA) burned [7]. Fox et al. examined CFD levels in 19 burn patients obtained during the first three days of hospitalization and after ten weeks. They found that CFD levels were significantly higher during the first 48 hours and related to TBSA in the first 24 hours [8].

Despite the discoveries mentioned above, CFD tests have not yet become routine, mainly due to the fact that current methods of CFD level analysis are impractical for routine clinical laboratory use. We have discovered a direct rapid fluorescent assay for CFD quantification that allows obtaining accurate and fast measurements that previously were obtained by the time-consuming and expensive quantitative polymerase chain reaction (PCR) technique [9]. This assay is based on the understanding that the SYBR Gold commercially available kit for qualitative detection of nucleic acids can be used in a quantitative way in a direct fluorometric assay. The purpose of this study was to implement this technique for obtaining CFD levels in burn patients and to preliminarily explore the use of CFD levels obtained by this direct fluorometric assay as a potential marker in burns, including the possibility of calculating a CFD level which could reflect survival outcome such as an LD_50_.

## 2. Methods

### 2.1. Cases and Controls

Fourteen burn victims admitted to the Soroka University Medical Center burn and intensive care units between March 2008 and May 2011 were studied. Inclusion criteria were otherwise healthy burn patients over 18 years old, brought directly by EMS to our medical center, within 6 hours of injury. Exclusion criteria were age under 18 years, admission later than 6 hours after injury, pregnancy, other concomitant trauma, or preexisting disease. Patient demographic and clinical data were recorded, including length of hospital stay, length of ICU stay, number of surgical operations, complete blood count variables, and blood chemistry panel variables. CFD levels of fourteen age- and gender-matched healthy volunteers served as a control group. The average age difference between the cases and controls was 0.3 years.

The study was approved by the Institutional Review Board.

### 2.2. CFD Analysis

Patient blood samples were obtained at admission in standard gel blood collection tubes (Vaccuette, Greiner Bio-One, Frickenhausen, Germany). Blood samples were centrifuged at 2000 G for 10 minutes at 4°C and serum was transferred to collection tubes and stored in −20°C. CFD levels were quantified by a direct rapid fluorometric assay, the fluorochrome SYBR Gold which does not require prior processing of samples, that is, DNA extraction and amplification [9]. Briefly, SYBR Gold Nucleic Acid Gel Stain (Invitrogen Paisley, UK) was diluted 1 : 1000 in dimethyl sulphoxide and then 1 : 8 in phosphate-buffered saline. Ten microliters of serum or DNA standard was applied to a 96-well plate and forty microliters of diluted SYBR Gold was applied to each well. Fluorescence was measured with a 96-well fluorometer (Spectrafluor Plus, Tecan, Durham, NC) at an emission wavelength of 535 nm and an excitation wavelength of 485 nm. The method was tested in comparison with the gold standard, QPCR, and was found to be in good correlation of *R* ^2^ = 0.9987 (*P* < 0.0001) as previously described [9].

### 2.3. Burn Assessment

Burn depth was clinically assessed by board-certified plastic surgeons experienced in burn care. TBSA was assessed by Lund and Browder burn charts. Total burn volume (TBV), a term that has not yet been coined in burn care, was calculated by multiplying TBSA (%) by the burn depth degree.

### 2.4. Statistical Analysis

Continuous variables are expressed as mean ± standard deviation (SD) and compared by Student's *t*-test. Due to the small amount of patients, we also used a-parametric tests (Mann-Whitney *U*). Categorical variables are expressed as proportions and compared with the *χ* ^2^ test. The correlations between variables were analyzed using Pearson's correlations, as well as Spearman's a-parametric correlation due to the small sample size. The level of significance was 0.05. LL_50_ was calculated as the CFD admission level above which 50% of patients did not survive. Analysis was performed using SPSS 18 System software (PASW, Chicago, IL).

## 3. Results

**Figure 1:**
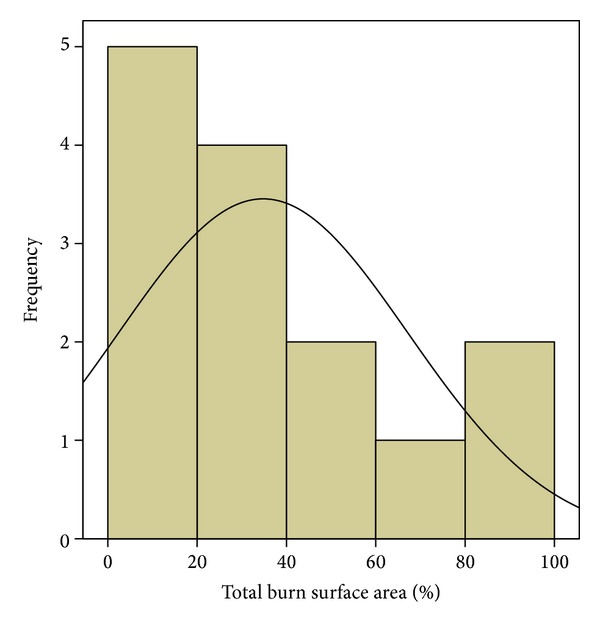
Total body surface area distribution among patients.

Demographic and clinical data for the 14 enrolled patients can be seen in Table 1. Four (29%) of the patients were female. The patients' mean age was 40 (±9.6) years. Twelve patients (86%) had burns due to fire, 1 (7%) due to electrocution, and 1 (7%) due to scald. Six patients (43%) suffered 2nd degree burns, 7 (50%) suffered 3rd degree burns, and 1 (7%) suffered 4th degree burns. The mean TBSA was 34.8% (±31.1%), and patient TBSA distribution can be seen in Figure 1. The mean number of hospitalization days was 21.3 (±20.2) days, ranging from 2 to 70 days. Ten patients (71%) were admitted to the intensive care unit (ICU), and their mean ICU treatment lasted 6.3 (±6.3) days. Four patients (29%) died during hospitalization.

We found no statistical difference between the cases and the controls considering gender (*χ* ^2^ = 0,  *P* = 1) or age (independent samples *t*-test = 0.77, *P* = 0.94).

The mean patient CFD level at admission was 1797 ng/mL (±1523 ng/mL) and the mean control group CFD level was 374 ng/mL (±245 ng/mL). This difference between cases and controls was found to be statistically significant (independent samples *t*-test = −3.452, *P* = 0.004) and was also proven in a-parametric analysis (M-W *U* = 186, *P* < 0.001).

**Figure 2:**
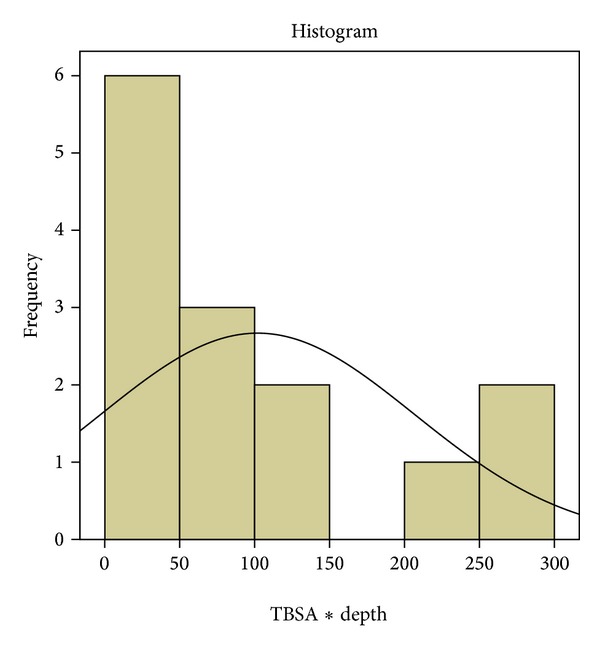
Total burn volume distribution among patients.

We examined the mathematical manipulation of multiplying the TBSA percentages by the burn depth degrees (TBSA% × burn degree) as a means of quantifying the total burn volume (TBV). TBV levels are shown in Figure 2.

**Figure 3:**
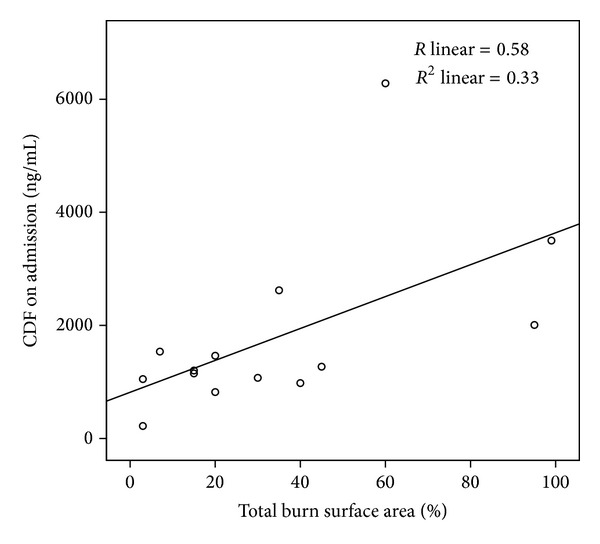
Correlation between admission cell free DNA (CFD) and total body surface area (*R* linear = 0.58).

**Figure 4:**
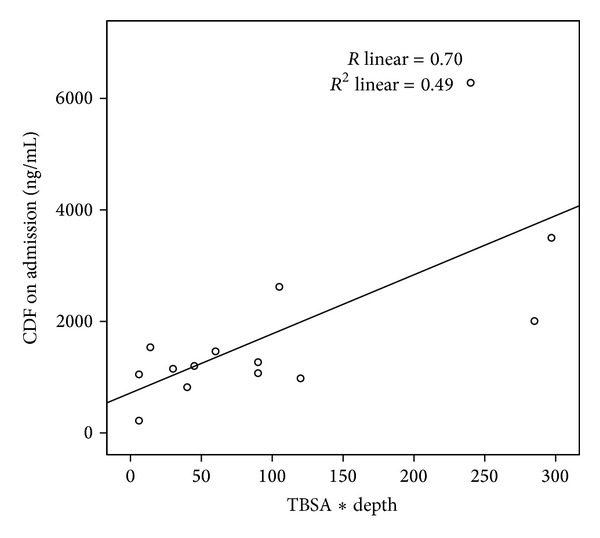
Correlation between admission cell free DNA (CFD) and total burn volume (*R* linear = 0.70).

Bivariate analyses show statistically significant correlations between CFD admission levels and burn degree, TBSA and TBV. Due to the small sample size, we used parametric and a-parametric correlations (Table 2). Graphs that depict correlations between admission CFD and TBSA (*R* = 0.58, see Figure 3) and TBV (*R* = 0.7, see Figure 4) are shown.

We found a significant difference (MW = 36, *P* = 0.024) between the CFD admission levels of the patients who died (mean = 3264 ± 2215) and those who survived (mean = 1211 ± 614). According to our results, an admission CFD level of 1200 ng/mL can be seen as an LL_50_ (lethal level of admission CFD over which 50% of the patients died) since of the eight patients with admission levels higher than 1200 ng/mL four did not survive.

We did not find significant correlations between admission CFD levels and length of hospital stay, length of ICU stay, number of surgical operations, complete blood count variables, and blood chemistry panel variables.

## 4. Discussion

The successful management of complex major burns is one of the most demanding in modern medicine, yet useful prognostic markers are sparse. A prognostic factor should reflect the total burn severity, that is, the total burn volume (TBSA X burn depth) and associated injuries, such as inhalation or electrocution injury. The cutaneous area of the burn can be assessed by the standard Lund and Browder burn charts. On the other hand, clinical estimation of burn depth at admission time has been shown to be highly unreliable [10] and diagnostic criteria for inhalation injury are rather vague and subjective [11]. As previously described by Fox et al. [8], a direct marker of cellular injury can provide a single objective assessment of the severity of the burn injury as a global pathological process. Accurate assessment of burn severity could thus guide management and prediction of outcome. During the past years, there has been increasing interest in CFD levels as a potential marker for burn severity. Previous work with CFD as a potential marker in the field of burns, although fruitful, was done using expensive and time-consuming methods, impractical for routine clinical laboratory use [7, 8]. Our work is unique since it demonstrates the routine clinical applicability of measuring admission CFD levels as a prognostic factor, using a simple, fast, and inexpensive method. A single CFD level test using our method costs approximately 0.05 USD and takes only several minutes to perform. This is based on the fact that materials for the analysis cost approximately 5 USD per 96 blood tests that necessitate approximately 30-minute work time. In comparison, Chiu et al. state the QPCR method as taking between 90 and 180 minutes to perform [7]. In recent studies we found that CFD levels measured using our method efficiently reflect the severity of disease and the dimensions of damaged tissue in human and experimental animal models [12–15].

Previous studies have shown that burn victims' CFD levels taken within 24 hours of injury are significantly elevated when compared to controls [7, 8]. Human CFD has a short half-life of approximately 16 minutes in circulation [4]. In this study, we chose to measure CFD levels within a shorter duration of time from injury in order to give a more accurate estimation of the initial burn severity. Our results show that patient admission CFD levels taken within 6 hours of injury are significantly elevated compared with that of controls. Our results also show statistically significant correlations between CFD admission levels and burn degree and TBSA. The mathematical manipulation of multiplying the clinical assessment of TBSA (%) by the clinical assessment of burn degree (1–4) can serve as an indicator for the total amount of burnt necrotic cells, that is, the assessed total burn volume (TBV). Our results show a statistically significant correlation between admission CFD levels and TBV as well, and this correlation is stronger than the one between CFD and TBSA, pointing to the possibility that the idea of crudely quantifying the total burn volume is an initial step in the correct direction of understanding the true extent of damage done by the initial burn insult. We believe that this mathematical manipulation would need to be validated in a larger patient population or perhaps finely tuned in some manner to better reflect the total burn volume and we intend to do so in future studies.

Four of our patients died due to burn trauma. We found that their admission CFD levels were significantly higher than those of the surviving patients and, as previously described, an admission CFD level of 1200 ng/mL can be seen as the LL_50_, that is, the level of admission CFD over which 50% of the patients had a lethal outcome.

## 5. Study Limitations

Our study has several limitations. We performed a single center study on a relatively small patient cohort. This cohort is quite heterogeneous and includes five patients with a TBSA lower than 20%, arguably burns that are not considered major. In addition, there was a patient with an electric 4th degree burn. Another important limitation, similarly to other studies with nonspecific markers of tissue damage, is that the many factors may affect baseline and postinjury levels of CFD irrespective of severity of the injury. This is particularly important in human studies, where basal (preinjury) levels of CFD cannot be measured. We attempted to minimize this limitation by excluding patients with another concomitant trauma or preexisting disease. Timing of sample collection also may influence the results, and we attempted to minimize this limitation by including only the patients from whom we managed to obtain a blood sample within 6 hours of injury.

Despite the above stated limitations, we believe this study has shown that levels of admission CFD correlate with burn area, depth, and volume.

## 6. Conclusions

Admission CFD levels can serve as a prognostic factor in burns. Further studies including more patients are needed in order to further strengthen our findings. Such future studies may help in making the way for CFD to become a routinely used clinical prognostic factor in the treatment of burn patients. We believe our direct rapid fluorometric technique for CFD level analysis can play an important part in such future progress.

## Figures and Tables

**Table 1:** Patient demographic and clinical data.

Patient #	Gender	Age (yrs)	Burn cause	Burn degree	TBSA	Hospitalization days	Patient outcome
1	Female	21	Fire	2	3%	6	Discharged
2	Male	34	Fire	2	3%	5	Discharged
3	Female	49	Scald	2	15%	15	Discharged
4	Male	45	Fire	3	95%	5	Died
5	Male	41	Fire	3	40%	47	Discharged
6	Male	57	Fire	2	20%	38	Discharged
7	Female	47	Fire	2	45%	14	Died
8	Male	39	Fire	3	99%	2	Died
9	Male	35	Fire	3	20%	38	Discharged
10	Male	45	Fire	3	35%	70	Discharged
11	Female	45	Fire	3	15%	19	Discharged
12	Male	28	Electric	4	60%	6	Died
13	Male	45	Fire	3	30%	27	Discharged
14	Male	29	Fire	2	7%	7	Discharged

**Table 2:** Statistical correlations between admission CFD (cell free DNA) levels and burn degree, TBSA (total burn surface area), and TBV (total burn volume).

	TBSA	Burn degree	TBV
Admission CFD (Pearson's correlation)	0.58 (*P* = 0.031)	Not applicable	Not applicable
Admission CFD (Spearman's correlation)	0.61 (*P* = 0.02)	0.78 (*P* = 0.001)	0.64 (*P* = 0.014)

## References

[1] Rainer TH, Lam NY (2006). Circulating nucleic acids and critical illness. *Annals of the New York Academy of Sciences*.

[2] Swarup V, Rajeswari MR (2007). Circulating (cell-free) nucleic acids—a promising, non-invasive tool for early detection of several human diseases. *FEBS Letters*.

[3] Rhodes A, Wort SJ, Thomas H, Collinson P, Bennett ED (2006). Plasma DNA concentration as a predictor of mortality and sepsis in critically ill patients. *Critical Care*.

[4] Lo YMD, Rainer TH, Chan LYS, Hjelm NM, Cocks RA (2000). Plasma DNA as a prognostic marker in trauma patients. *Clinical Chemistry*.

[5] Gautschi O, Bigosch C, Huegli B (2004). Circulating deoxyribonucleic acid as prognostic marker in non-small-cell lung cancer patients undergoing chemotherapy. *Journal of Clinical Oncology*.

[6] Saukkonen K, Lakkisto P, Pettilä V (2008). Cell-free plasma DNA as a predictor of outcome in severe sepsis and septic shock. *Clinical Chemistry*.

[7] Chiu TW, Young R, Chan LYS, Burd A, Lo DYM (2006). Plasma cell-free DNA as an indicator of severity of injury in burn patients. *Clinical Chemistry and Laboratory Medicine*.

[8] Fox A, Gal S, Fisher N (2008). Quantification of circulating cell-free plasma DNA and endothelial gene RNA in patients with burns and relation to acute thermal injury. *Burns*.

[9] Goldshtein H, Hausmann MJ, Douvdevani A (2009). A rapid direct fluorescent assay for cell-free DNA quantification in biological fluids. *Annals of Clinical Biochemistry*.

[10] Pape SA, Skouras CA, Byrne PO (2001). An audit of the use of laser Doppler imaging (LDI) in the assessment of burns of intermediate depth. *Burns*.

[11] Clark CJ, Reid WH, Gilmour WH, Campbell D (1986). Mortality probability in victims of fire trauma: revised equation to include inhalation injury. *British Medical Journal*.

[12] Boyko M, Ohayon S, Goldsmith T (2011). Cell-free DNA-A marker to predict ischemic brain damage in a rat stroke experimental model. *Journal of Neurosurgical Anesthesiology*.

[13] Shimony A, Zahger D, Gilutz H (2010). Cell free DNA detected by a novel method in acute ST-elevation myocardial infarction patients. *Acute Cardiac Care*.

[14] Czeiger D, Shaked G, Eini H (2011). Measurement of circulating cell-free DNA levels by a new simple fluorescent test in patients with primary colorectal cancer. *American Journal of Clinical Pathology*.

[15] Ohayon S, Boyko M, Saad A (2012). Cell-free DNA as a marker for prediction of brain damage in traumatic brain injury in rats. *Journal of Neurotrauma*.

